# The effects of CRISPR-Cas9 knockout of the TGF-β1 gene on antler cartilage cells in vitro

**DOI:** 10.1186/s11658-019-0171-z

**Published:** 2019-06-22

**Authors:** Mingxiao Liu, Xiangyu Han, Hongyun Liu, Danyang Chen, Yue Li, Wei Hu

**Affiliations:** College of Life Sciences, Jilin Agriculture University, Changchun, 130118 Jilin Province China

**Keywords:** CRISPR-Cas9, Sika deer, Transforming growth factor-β1, Cartilage cells

## Abstract

**Background:**

Deer antler is the only mammalian organ that can be completely regenerated every year. Its periodic regeneration is regulated by multiple factors, including transforming growth factor β (TGF-β). This widely distributed multi-functional growth factor can control the proliferation and differentiation of many types of cell, and it may play a crucial regulatory role in antler regeneration. This study explored the role of TGF-β1 during the rapid growth of sika deer antler.

**Methods:**

Three CRISPR-Cas9 knockout vectors targeting the TGF-β1 gene of sika deer were constructed and packaged with a lentiviral system. The expression level of TGF-β1 protein in the knockout cell line was determined using western blot, the proliferation and migration of cartilage cells in vitro were respectively determined using EdU and the cell scratch test, and the expression levels of TGF-β pathway-related genes were determined using a PCR array.

**Results:**

Of the three gRNAs designed, pBOBI-gRNA2 had the best knockout effect. Knockout of TGF-β1 gene inhibits the proliferation of cartilage cells and enhances their migration in vitro. TGF-β signaling pathway-related genes undergo significant changes, so we speculate that when the TGF-β pathway is blocked, the BMP signaling pathway mediated by BMP4 may play a key role.

**Conclusions:**

TGF-β1 is a newly identified regulatory factor of rapid growth in sika deer antler.

## Introduction

Deer antler, which is a secondary male sexual characteristic in most species of deer, is the only mammalian organ that can completely regenerate each year [1, 2]. Antler tissue has a very rapid growth rate, reaching up to 2 cm per day at its maximum, and on average,10 kg of bone tissue grows in about 60 days [3, 4]. Surprisingly, antler growth always occurs in an orderly manner under the guidance of morphogenetic information with no recorded occurrence of cancer [5]. For this reason, deer antler has deservedly become a biological model of great value in various fields.

The development and regeneration of deer antler are associated with a variety of small biomolecules, including insulin-like growth factor (IGF), bone morphogenetic protein (BMP), epidermal growth factor (EGF), nerve growth factor (NGF), fibroblast growth factor (FGF), vascular endothelial growth factor (VEGF) and transforming growth factor (TGF) [6, 7]. These growth factors play important roles through autocrine and paracrine pathways, and their activities are closely related to the regulation of antler regeneration [8].

TGF-β is a multi-functional cytokine that regulates cell proliferation, cell differentiation and extracellular matrix production. It is of great significance in development, wound healing, organ fibrosis and tumor metastasis [9]. TGF-β has three subtypes in mammals: TGF-β1, TGF-β2 and TGF-β3 [10]. Activated TGF-β ligands bind to TGF-β receptors on the cell surface and rely on the formation of ligand–receptor complexes to initiate signal transmission inside the cell. This leads to the activation of Smad proteins and ultimately causes a nuclear factor response [11].

Clustered regularly interspaced short palindromic repeats (CRISPRs) are a form of acquired immune system induced by RNA. The CRISPR immune defense mechanism evolved from bacteria and archaea, where it defends against the continuous attack of viruses and plasmids [12–14].

The CRISPR system recognizes DNA through base pairing based on a pair of RNAs. It directs CRISPR-associated nuclease 9 (Cas9) to cleave the double-stranded DNA that it recognizes, resulting in a double-strand break (DSB) [15]. The formation of a DSB induces the cell’s own repair mechanism, which includes non-homologous end joining (NHEJ) and homology-directed repair (HDR). Thus, the editing of the target DNA is ultimately achieved [16, 17].

Cas9 is a naturally-occurring endonuclease with two enzyme cleavage domains: the HNH nuclease domain and the Ruv-C-like domain. They respectively cleave the complementary and non-complementary strands. In the cutting process, two assistant RNAs are needed for guidance in bacteria: CRISPR RNA (crRNA) and trans-activating crRNA (tracrRNA) [18, 19]. The function of these two RNAs can be achieved with synthetic single-guide RNA (gRNA) now, which is sufficient to guide Cas9 to achieve site-directed cleavage.

Our previous immunohistochemistry results have confirmed that TGF-β1 was expressed in the skin, mesenchymal and cartilage layers of sika deer antler. The highest relative expression level was in the cartilage layer. In this study, we used CRISPR-Cas9 to knock out the TGF-β1 gene of antler cartilage cells, allowing the investigation of the effect of TGF-β1 on the growth and regeneration of sika deer antler.

## Materials and methods

### Exon prediction and gRNA design

Since the whole genome sequence of sika deer has not been released, near-source species were used for gRNA design. A partial sequence of sika deer TGF-β1 was cloned via RT-PCR. The TGF-β1 sequences of pig (*Sus scrofa*, NM_214015.2), goat (*Capra hircus*, NC_030825.1) and cattle (*Bos taurus*, NM_001166068) with high homology were obtained via blast alignment. The alignment of the three sequences showed that the positions of the exons and introns are substantially the same and the exons of sika deer TGF-β1 were predicted by referring to the three sequences.

The three gRNA sequences were designed and synthesized using the CRISPR online design tool at http://crispr.mit.edu/. Based on those results, three sequences with higher scores in the first exon were selected to design the CRISPR oligonucleotide chains.

### Lentiviral packaging

The three pairs of annealed gRNAs were ligated to the pBOBI vector. The recombinant plasmids that were positive for sequencing were named pBOBI-gRNA1, pBOBI-gRNA2 and pBOBI-gRNA3. After that, three plasmids (pMDL, VSV-G, REV) required for lentiviral packaging were transfected into *E. coli*-competent cells (DH5α) and the plasmids were extracted using an Endotoxin-free plasmid kit. Then, the lentiviruses were produced via packaging in 293 T cells.

When the cells reached a growth density of 80%, we proceeded with calcium phosphate transfection. First, we prepared a lentiviral packaging plasmid mixture in the ratio pMDL:VSV-G:REV = 5:3:2, with a total concentration of 1 μg/μL. Second, we took two clean EP tubes (designated A and B). We added 400 μL 2 × HBS, 5 μg recombinant plasmid and 5 μg packing plasmid mixture into tube A and mixed. Simultaneously, we added an equal volume 2 × CaCl_2_ into tube B. Then we added the solution in tube B dropwise to tube A and incubated it for 10 min at room temperature. Finally, the transfection complex was slowly and uniformly added into 293 T cells and incubated at 37 °C for 12 h. The medium was changed for Dulbecco’s modified Eagle medium (DMEM) after 12 h, and virus supernatants were collected twice at 48 and 72 h.

### Cell culture

Deer antler samples were obtained from a three-year old male sika deer (*Cervus nippon*) provided by the Jilin Agricultural University Deer Farm in Changchun, China. Cartilage tissues were isolated under a dissecting microscope. The tissues were digested with collagenase-I and hyaluronidase for 1.5 h at 37 °C, after which they were digested with collagenase-II for 3 h under the same conditions. After centrifugation, the cartilage cells were cultured in DMEM supplemented with 20% (v/v) fetal bovine serum (FBS), 200 U/mL penicillin and 100 U/mL streptomycin at 37 °C with 5% (v/v) CO_2_. This study was approved by the Ethical Committee for Laboratory Animals at Jilin Agricultural University (permit no. ECLA-JLAU-17031).

### Cell infection

Cartilage cells were seeded into 12-well plates at a density of 10^5^ cells/mL, then we added 2 mL DMEM to each well for overnight growth. When the cells reached a growth density of more than 70%, they were infected with GFP-expressing lentivirus using different MOI gradients (MOI = 1, 3, 5), and observed using fluorescence microscopy to obtain the optimal MOI of antler cartilage cells.

Next, the cells of each experimental group were infected with the optimal MOI, the polybrene was added at a final concentration of 8 μg/mL to increase the infection efficiency. After infection for 24 h, we changed the medium containing lentivirus for fresh complete medium and continued the culture at 37 °C. The medium was changed to G418-containing medium after 48 h. An equal concentration of G418 was added to wild-type cells as the control. We changed the medium every 2–3 days until the control cells were all dead. The stable knockout cell lines were thus obtained.

### Western blot analysis

The total proteins of the knockout cell lines were extracted, and the concentration of the collected proteins was determined using the protein assay kit via the BCA method. The relative expression level of TGF-β1 protein in the three knockout cell lines was detected via western blot to determine the gene knockout effect. After mixing with loading buffer, the protein was separated using 12% SDS-PAGE and transferred to PVDF membranes. Non-specific binding was blocked with 5% evaporated milk, and then the membranes were incubated with primary antibodies of TGF-β1 and GAPDH (Bioss) at 4 °C overnight. After that, the membranes were incubated with the secondary antibodies at room temperature for 2 h. After washing with TBST three times, the signals were detected using enhanced chemiluminescence (ECL) reagents and the protein band intensities were analyzed using Image-Pro Plus software. In this experiment, untreated cells were used as the normal control group, GFP-infected cells were used as the negative control group, and GAPDH was used as the internal reference.

### Cell proliferation assays

Cartilage cells were seeded into 96-well plates at a density of 4000 cells per well. We added 200 μL DMEM to each well for overnight growth. The cell proliferation of untreated group, negative control group and experimental group were examined after 24, 48 and 72 h. Cartilage cells were stained with EdU and Hoechst 33342 and cell proliferation was then evaluated using fluorescence microscope.

### Detection of cartilage cell migration

First, we drew 3 uniform horizontal lines on the back of 12-well plates. Then, the transfected cells were seeded in the plates, giving full coverage, and incubated overnight. The next day, we used a pipette tip to draw three straight lines in vertical to the lines on the backs of the plates. Separated cells were washed away with PBS and cultured in serum-free medium for 24 h. We took pictures at 0, 6, 12 and 24 h.

### PCR array assays

Total RNA was extracted from the knockout cell lines using the Trizol method. RNA purity and concentration were determined using the ultraviolet absorption method. The integrity of the RNA band was detected via capillary electrophoresis.

First, the total RNA was reverse transcribed to cDNA. The reagents in the RT^2^ SYBR Green Mastermix tube were centrifuged to the bottom of the tube and the PCR reaction mixture was prepared: 1350 μL of 2 × RT^2^ SYBR Green Mastermix, 102 μL of cDNA solution and 1248 μL of RNase-free water. Then, we added 25 μL of PCR reaction mixture into each well of RT^2^ Profiler PCR Array for real-time quantitative PCR. The procedure was 40 cycles of: 95 °C for 1 min, 95 °C for 15 s and 60 °C for 1 min. The expression levels of the TGF-β pathway-related genes in the control and experimental group were analyzed with heat maps and scatter plots.

### Statistical analysis

All the experimental data are presented as means ± standard deviation (S.D.). SPSS 22.0 software was used for all the statistical analysis. The significance of differences between groups was evaluated using Student’s t test. *p* < 0.05 was considered statistically significant.

## Results

### Exon prediction and gRNA design

**Fig. 1:**
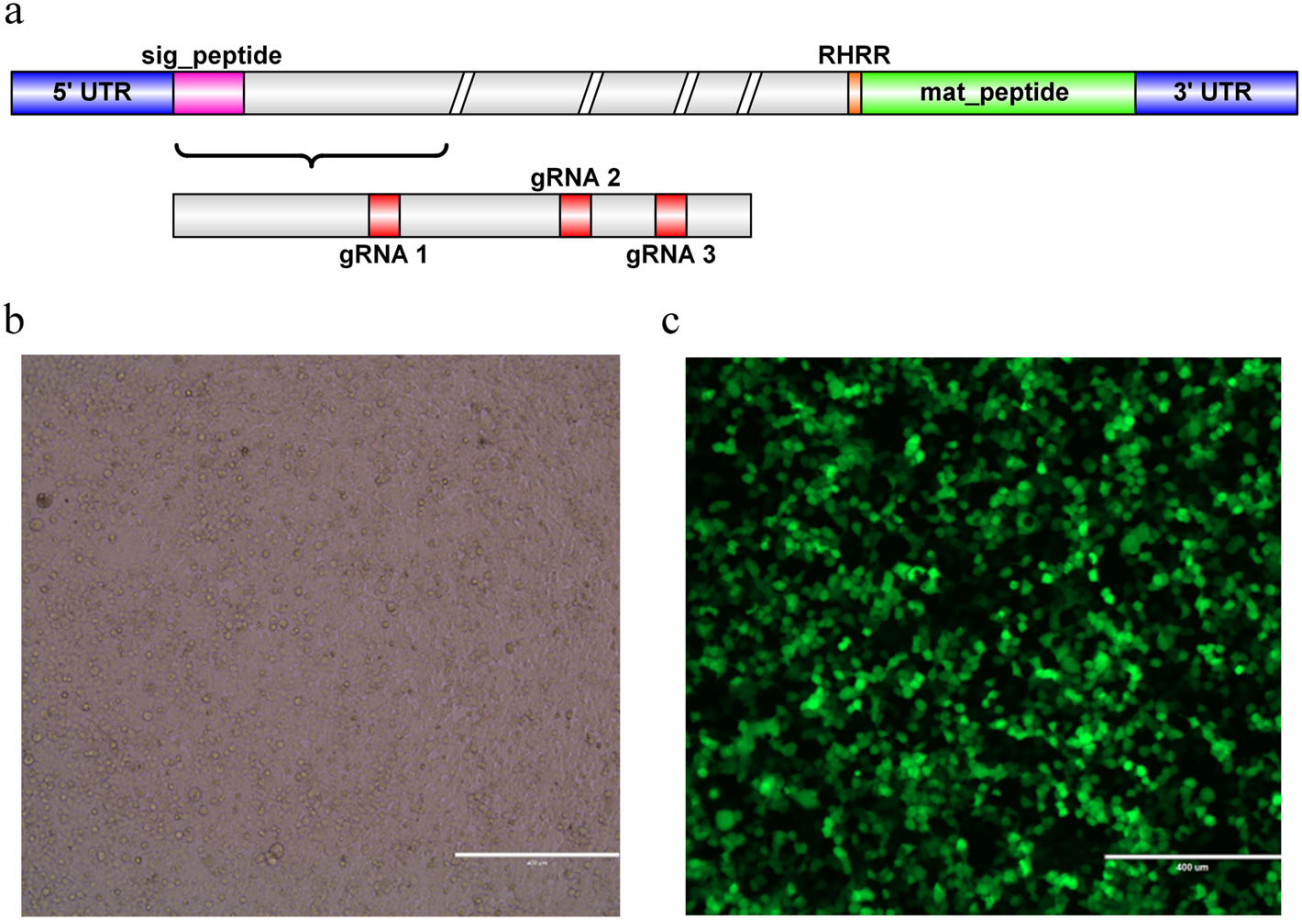
gRNA design and lentiviral packaging. **a** – The exons of sika deer TGF-β1 were analyzed using blast alignment of related sequences, and three gRNA oligonucleotide sequences were designed at the first exon. **b** – Visible light image of GFP protein. **c** – Fluorescence image of GFP protein

We divided the exons of TGF-β1 and designed three gRNAs at the first exon (Fig. 1a). The PAM sequence of the oligonucleotide strand was removed and a restriction enzyme cleavage site was added at both ends, i.e., we added CACCG bases to the 5′ end of coding strand and AAAC bases to the 5′ end of non-coding strand (Table 1). Finally, the three synthetic CRISPR oligonucleotide strands were annealed and ligated into the pBOBI vector. The sequencing results indicate that the three recombinant plasmids were successfully constructed.

**Table 1:** gRNA sequences

Name	Sequence (5′ to 3′)
gRNA1-F	caccgGGTGAAGCGGAAGCGCATCG
gRNA1-R	aaacCGATGCGCTTCCGCTTCACCc
gRNA2-F	caccgTTTACAACAGTACCCGCGAC
gRNA2-R	aaacGTCGCGGGTACTGTTGTAAAc
gRNA3-F	caccgTTGGCGTAGTAGTCCGCCTC
gRNA3-R	aaacGAGGCGGACTACTACGCCAAc

### Lentiviral packaging

The 293 T cells were infected with the collected lentivirus, and the expression of GFP protein after 48 h was observed via fluorescence microscopy (Fig. 1b and c). The results showed successful lentiviral packaging of the CRISPR-Cas9 knockout vector. The virus titer was higher than 10^8^ TU/mL.

### Relative expression of TGF-β1 protein

**Fig. 2:**
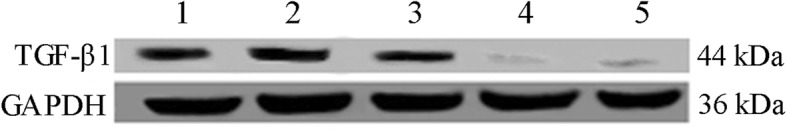
Relative expression levels of TGF-β1 protein. (1) Untreated group. (2) Negative control group. (3) pBOBI-gRNA1 infected group. (4) pBOBI-gRNA2 infected group. (5) pBOBI-gRNA3 infected group

The relative expression level of TGF-β1 protein was determined via western blot (Fig. 2), and the experimental results were analyzed using Image-Pro Plus (Table 2). The results show that the expression of TGF-β1 protein was lowest in pBOBI-gRNA2 infected group, indicating that the greatest knockout effect was for gRNA2.

**Table 2:** Densitometric analysis of TGF-β1 protein expression levels

	Untreated	Negative control	gRNA1	gRNA2	gRNA3
TGF-β1	122.45 ± 0.27	177.34 ± 0.56	126.01 ± 0.39	2.37 ± 0.16	5.19 ± 0.13
GAPDH	635.32 ± 0.83	679.98 ± 0.26	658.72 ± 0.76	654.75 ± 0.28	626.64 ± 0.68
Homogenization	99.26 ± 0.34	134.31 ± 0.40	98.52 ± 0.30	1.87 ± 0.13	4.26 ± 0.12

### EdU assays

**Fig. 3:**
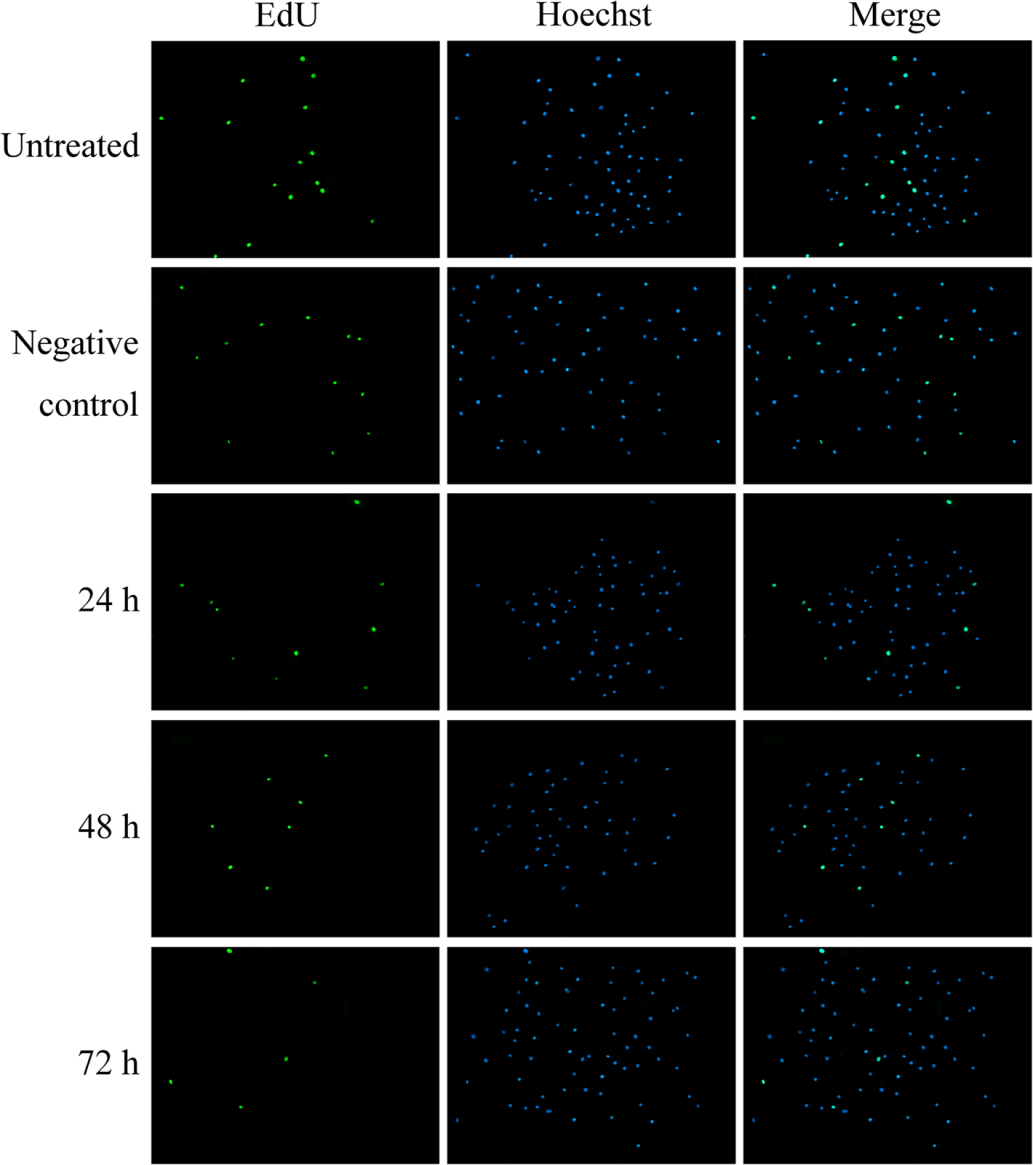
EdU detection of cartilage cells proliferation (×100)

The cartilage cells were infected with pBOBI-gRNA2 lentivirus, and the proliferation of each experimental group was detected using the EdU method after culture for 24, 48 and 72 h. The proliferation of knockout cell was significantly lower than for the control group, and cartilage cell proliferation remained inhibited over time, indicating that the knockout of TGF-β1 can affect the proliferation of cartilage cells in vitro (Fig. 3).

### Detection of cartilage cell migration

**Fig. 4:**
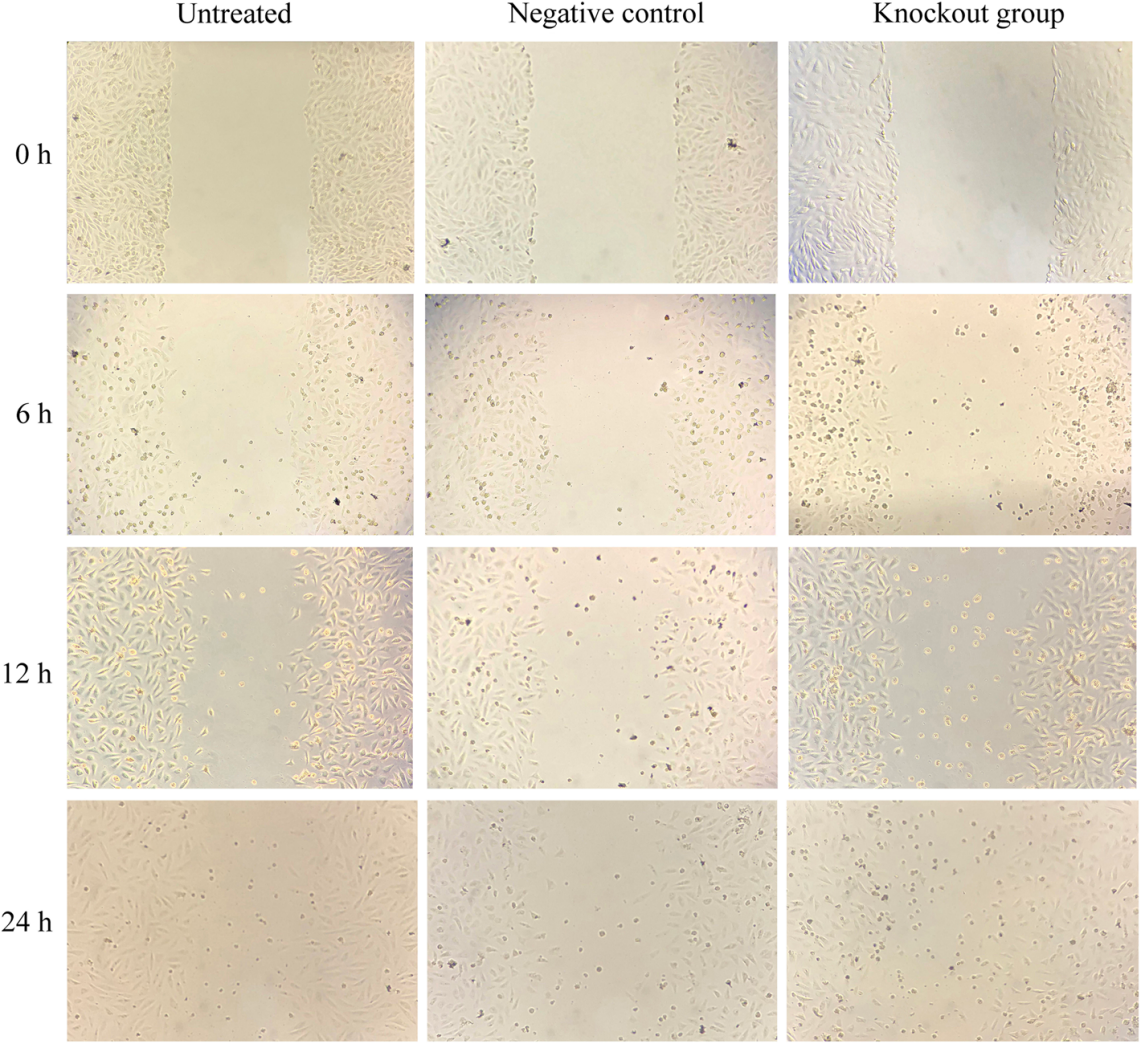
Cell scratch assay for cartilage cell migration (× 100)

The changes in cartilage cell migration in the untreated, negative control and experimental groups were observed at 0, 6, 12 and 24 h. The results show that the cell migration in the experimental group was significantly higher than that in the other two groups, and no significant difference between the untreated and negative control groups appeared over time (Fig. 4). This shows that TGF-β1 knockout may promote the cartilage cell migration in vitro.

### PCR array results

**Fig. 5:**
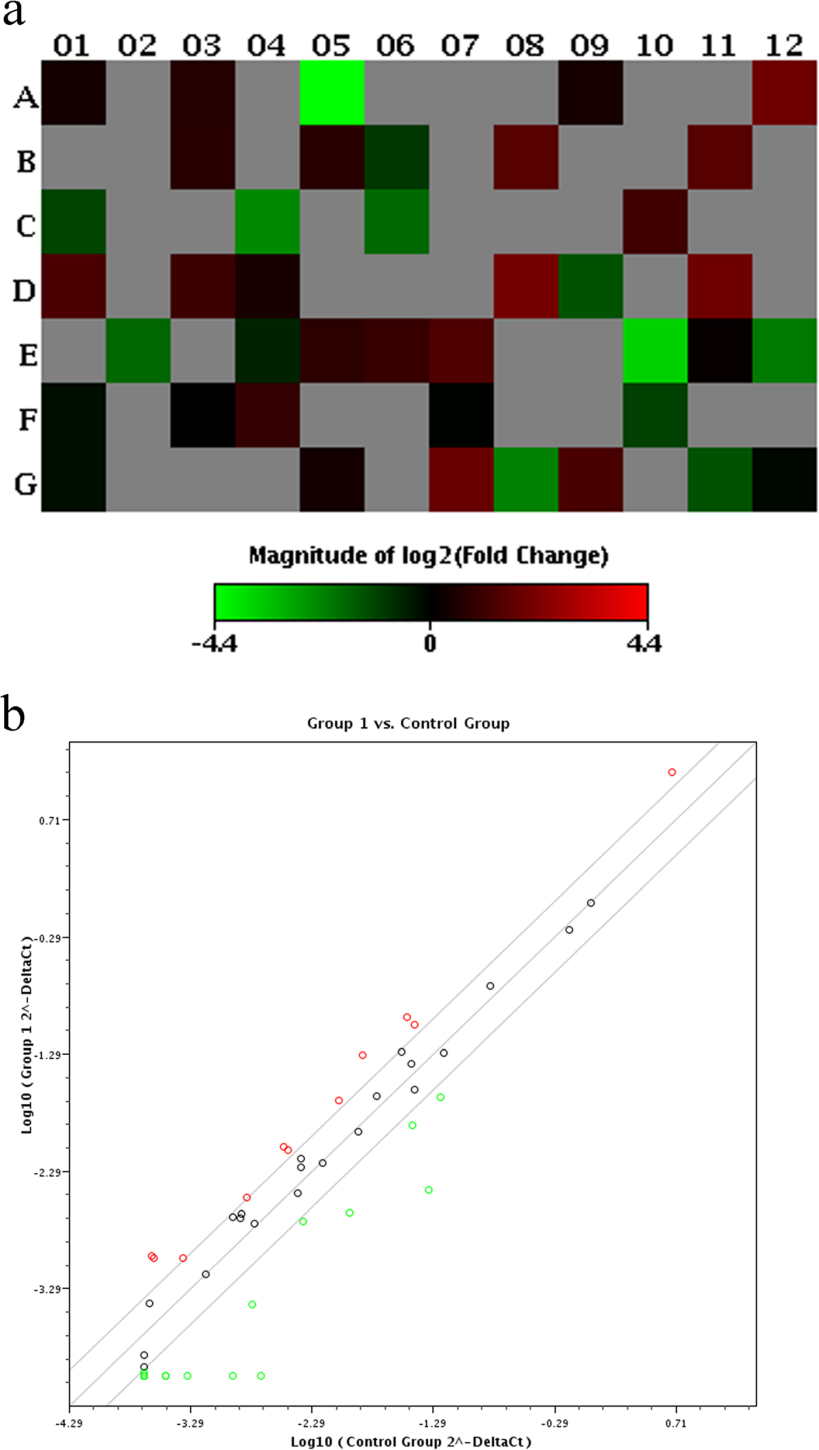
Results of PCR array. **a** – Heat map analysis of the relationship between TGF-β1 knockout and TGF-β pathway-related gene expression. **b** – Scatter plot analysis of the relationship between TGF-β1 knockout and TGF-β pathway-related gene expression

In our PCR array experiment, untreated cartilage cells were used as the control, and TGF-β1 knockout cell lines were used as experimental groups. The heat map (Fig. 5a) and scatter plot (Fig. 5b) analyses indicate that the knockout of the TGF-β1 gene led to the upregulation of 11 related genes and the downregulation of 9 related genes in the pathway (Tables 3 and 4). Among them, we found several key genes that have changed. For example, BMP4 and ID2 were upregulated and BMPR2 and Smad1 were downregulated.

**Table 3:** Upregulated genes in TGF-β1 knockout cell lines

Position	Gene symbol	Fold regulation
D08	LTBP1	3.9724
A12	BMP4	3.7581
D11	NOG	3.7064
G07	PMEPA1	3.4343
B08	CDKN1B	2.7895
B11	COL1A1	2.7702
E07	SMAD7	2.5315
D01	ID2	2.4284
G09	SMAD6	2.362
C10	GSC	2.1287
D03	IGF1	2.0562

**Table 4:** Downregulated genes in TGF-β1 knockout cell lines

Position	Gene symbol	Fold regulation
A05	AMHR2	−21.1121
E10	TGFB1	−12.295
C04	GADD45B	−5.1694
G08	SERPINE1	−4.6268
E12	TGFB2	−4.3469
C06	GDF3	−3.4343
E02	SMAD1	−3.4105
G11	STK38L	−2.6945
F10	BMPR2	−2.1287

## Discussion

Deer antler has a stable growth cycle, grows with a fixed shape and position, and can be completely regenerated every year, making it an ideal mammalian morphogenesis model [20]. Previous studies have shown that deer antler derives from the proliferation and differentiation of antlerogenic periosteum (AP) stem cells [21]. When the first antler undergoes shedding, its morphological information transfers from the AP to the pedicle periosteum (PP). This is accompanied by the disappearance of the AP and permanent retention of the PP [22]. The PP stem cells differentiate and regenerate to form a complete antler the following year.

Activation of AP and PP cells is primarily regulated by androgens, which stimulate them to produce a large quantity of cell growth factors that stimulate rapid antler growth [23]. Among them is TGF-β, which regulates cell proliferation, cell differentiation, extracellular matrix production and other processes [24, 25].

It is worth mentioning that the regulation of cell proliferation by TGF-β is particularly complicated. TGF-β may have different effects in different cell types. Also, for the same type of cells, TGF-β has a bidirectional regulation effect due to different stimulation doses [26].

To further study the effect of TGF-β1 on antler regeneration and proliferation, we constructed three CRISPR-Cas9 knockout vectors targeting the TGF-β1 gene of sika deer. They were packaged using a lentiviral system, and we used lentivirus infection and G418 resistance screening to obtain a TGF-β1 knockout cell line of cartilage cells. The expression level of TGF-β1 protein in the knockout cell line was detected using western blot. pBOBI-gRNA2 showed the strongest and most stable knockout of TGF-β1, which indicated the second knockout site we designed is suitable. Then, we examined the effect of TGF-β1 on the proliferation and migration of cartilage cells in vitro. The results show that the lack of TGF-β1 inhibited the proliferation of cartilage cells in vitro, but promoted the migration of cartilage cells, which is of great significance to cartilage cells in sika deer.

The influence of TGF-β1 on the expression level of TGF-β signaling pathway-related genes was detected using a PCR array. It showed that the knockout of the TGF-β1 gene resulted in both the upregulation and the downregulation of various related genes in the pathway. Further analysis revealed that the knockout of TGF-β1 mainly resulted in the upregulation of BMP4 and ID2 and the downregulation of BMPR2 and Smad1. Of these, ID2 is one of the most important target genes regulated by BMPs. It can be used as a negative or positive regulator of cell differentiation. BMPR2 and Smad1 are downstream molecules of BMP signaling. We speculate that when the TGF-β pathway is blocked, the BMP signaling pathway mediated by BMP4 may play a key role, and the specific mechanism needs further verification and exploration.

## Conclusions

Our research shows that TGF-β1 is a crucial regulatory factor in antler cartilage cells. It causes a decrease in cell proliferation and an increase in migration. It also activates the transmission of the BMP4 signaling pathway. However, the specific mechanism that mediates antler regeneration requires further study.

## Data Availability

The datasets used and/or analyzed in this study are available from the corresponding author on reasonable request.

## Abbreviations

BMP: Bone morphogenetic protein
Cas9: CRISPR-associated nuclease 9
CRISPR: clustered regularly interspaced short palindromic repeats
crRNA: CRISPR RNA
DSB: Double-strand break
EGF: Epidermal growth factor
FGF: Fibroblast growth factor
gRNA: Single-guide RNA
HDR: Homology-directed repair
IGF: Insulin-like growth factor
NGF: Nerve growth factor
NHEJ: Non-homologous end joining
TGF-β: Transforming growth factor β
tracrRNA: trans-activating crRNA
VEGF: Vascular endothelial growth factor
